# A Case of GCH-1 Mutation Dopa-Responsive Dystonia Requiring High Doses of Levodopa for Treatment

**DOI:** 10.5334/tohm.619

**Published:** 2021-06-24

**Authors:** Maeve Bradley, Shane Lyons, Sarah Coleman, Roisin Vance, Fiona Molloy, Peter Widdess-Walsh

**Affiliations:** 1 Department of neurology, Beaumont Hospital, Dublin; 2 Department of physiotherapy, Beaumont Hospital, Dublin

**Keywords:** GCH-1 mutation, dopa-responsive, dystonia, levodopa, segawa

## Abstract

**Background:**

Mutations in the GCH-1 gene are associated with Autosomal Dominant Dopamine Responsive Dystonia (DYT 5). One of the hallmarks of this condition is dramatic and sustained response to low doses of levodopa.

**Case Report:**

We present the case of a 22 year old female patient with genetically confirmed GCH-1 Dopa-Responsive Dystonia who had no response to low dose Levodopa but who achieved symptom control on a total dose of 900 mg/day.

**Discussion:**

Autosomal Dominant Dopa-Responsive Dystonia is a phenotypical heterogenous condition that, in some cases, may require high doses of levodopa for treatment response.

**Highlights:**

Mutations in the GCH-1 gene are associated with Autosomal Dominant Dopamine Responsive Dystonia which is typically defined by dramatic responses to low doses of levodopa. We report a patient with genetically confirmed Dopa-Responsive Dystonia who had no response to low dose Levodopa but who achieved symptom control with 900 mg/day.

## Introduction

Autosomal Dominant Dopa-Responsive Dystonia (DRD) is a phenotypically heterogenous condition typically presenting in childhood or adolescence with focal dystonia, diurnal fluctuations and occasionally mild parkinsonism. Mutations in the GCH-1 gene encoding the enzyme GTP cyclohydrolase are the most common cause [1]. A hallmark of this condition is marked and sustained responses to low dose levodopa. This makes it an essential condition to recognize as symptoms are potentially treatable and treatment associated dyskinesias are uncommon [2]. We present a case of DRD with a pathogenic confirmed GCH-1 mutation who had no treatment response to low doses of levodopa but achieved complete symptoms control on a higher dose (900 mg/day).

## Case Report

A 22 year old right handed female was referred to Neurology outpatients with a three year history of lower limb cramping relieved by movement and involuntary nocturnal lower limb movements. She was able to suppress the movements but this increased discomfort. Initially, there was a brief and non-sustained response to a dopamine agonist (pramipexole commencing at 0.18 mg daily up titrated to 2 g three times a day). In addition, she reported painful involuntary pulling sensation in her head and trunk. Past medical history was unremarkable and there was no history of Dystonia or Parkinsonism in her two sisters, parents or grandparents. No identifiable triggers including trauma or neuroleptic use were identified. Over the next two months, these symptoms rapidly progressed and the patient was admitted for assessment. Examination at that time revealed hyperkinetic movements comprising of extensor posturing in the upper limbs, severe retrocollis and retropulsion of the neck and torso. There was no notable diurnal variation. There was no evidence of Parkinsonism, pyramidal or cerebellar dysfunction. Deep tendon reflexes were brisk and symmetrical with downgoing plantars. There was no clinical response during a period of direct observation with inpatient neurology and physiotherapy assessment to a low dose of Carbidopa-Levodopa (Sinemet) 10 mg/100 mg three times a day (TID). Her symptoms continued to progress and two months later, the patient was unable to mobilise independently or lie flat.

A Dystonia and Parkinsonism Next Generation Sequencing panel was requested and revealed a heterozygous point mutation GCH1gene c.305T>A with a Met102Lys protein change. This mutation has previously been reported with Autosomal Dominant Dopa-Responsive Dystonia [3,4,5]. CSF biomarkers revealed low levels of biotetrahypterin and total neopterin which supported a defect in GTP-cyclohydrolase. She was retrialled on a higher dose of Carbidopa-Levodopa (Sinemet) 30 mg/300 mg TID with complete resolution of symptoms (total daily levodopa dose 900 mg). At two year follow up, the patient remains asymptomatic on the same dose. The medication is well tolerated without adverse effects and the patient has an excellent quality of life.

## Discussion

Dopa-Responsive Dystonia was first described by Masaya Segawa in 1976 [2]. A defect in GCH-1 gene is the most common cause, and results in defects in the enzyme GTP-cyclohydrolase [1]. This enzyme catalyses the first step in biosynthesis of Biotetrahypterin, a cofactor for Tyrosine Hydroxylase. The classic syndrome associated with this enzyme deficiency presents in the first decade of life with foot dystonia [6]. Diurnal fluctuation is common with symptoms worsening during the day. Brisk reflex and clonus can also been seen. Later on in the disease course, Parkinsonism can develop. Postural instability and retropulsion have been reported [6]. Most patients have a marked beneficial response to Levodopa at low doses and treatment associated dyskinesias are uncommon.

This case demonstrates many atypical features including; dystonic phenomenology at late age onset, lack of diurnal variation and lack of family history and importantly, lack of response to low dose levodopa. However, case reports do show a wide variation in clinical syndromes associated with this condition but to our knowledge the high levodopa requirements are unique to this case.

Three international studies have reported families with the same point mutation with amino acid change Methionine Lysine 102 as seen in this case [3,4,5]. The reports are phenotypically heterogenous even within the same families. However, all patients had an excellent response to Levodopa at doses 25–400 mg/day [3,4,5]. DYT5 is an important cause of treatable dystonia. If clinical suspicion is high despite lack of response to low dose levodopa, higher doses of levodopa may produce a clinical response, and significantly improve quality of life for these patients.
